# Adaptation Mechanisms in the Evolution of Moss Defenses to Microbes

**DOI:** 10.3389/fpls.2017.00366

**Published:** 2017-03-15

**Authors:** Inés Ponce de León, Marcos Montesano

**Affiliations:** ^1^Departamento de Biología Molecular, Instituto de Investigaciones Biológicas Clemente EstableMontevideo, Uruguay; ^2^Laboratorio de Fisiología Vegetal, Centro de Investigaciones Nucleares, Facultad de Ciencias, Universidad de la RepúblicaMontevideo, Uruguay

**Keywords:** moss-microbe interactions, pathogens, adaptation mechanisms, evolution, plant defenses, horizontal gene transfer

## Abstract

Bryophytes, including mosses, liverworts and hornworts are early land plants that have evolved key adaptation mechanisms to cope with abiotic stresses and microorganisms. Microbial symbioses facilitated plant colonization of land by enhancing nutrient uptake leading to improved plant growth and fitness. In addition, early land plants acquired novel defense mechanisms to protect plant tissues from pre-existing microbial pathogens. Due to its evolutionary stage linking unicellular green algae to vascular plants, the non-vascular moss *Physcomitrella patens* is an interesting organism to explore the adaptation mechanisms developed in the evolution of plant defenses to microbes. Cellular and biochemical approaches, gene expression profiles, and functional analysis of genes by targeted gene disruption have revealed that several defense mechanisms against microbial pathogens are conserved between mosses and flowering plants. *P. patens* perceives pathogen associated molecular patterns by plasma membrane receptor(s) and transduces the signal through a MAP kinase (MAPK) cascade leading to the activation of cell wall associated defenses and expression of genes that encode proteins with different roles in plant resistance. After pathogen assault, *P. patens* also activates the production of ROS, induces a HR-like reaction and increases levels of some hormones. Furthermore, alternative metabolic pathways are present in *P. patens* leading to the production of a distinct metabolic scenario than flowering plants that could contribute to defense. *P. patens* has acquired genes by horizontal transfer from prokaryotes and fungi, and some of them could represent adaptive benefits for resistance to biotic stress. In this review, the current knowledge related to the evolution of plant defense responses against pathogens will be discussed, focusing on the latest advances made in the model plant *P. patens*.

## Interaction of Basal Land Plants with Microbes

Bryophytes, non-vascular plants including mosses, liverworts, and hornworts are early evolutionary clades, which were among the first plants that colonized land. These plants acquired adaptation mechanisms to cope with different kinds of abiotic stresses, including variations in temperature, UV-B radiation, desiccation stress (Rensing et al., 2008), as well as defenses to pre-existing microorganisms (Raven, 1984). A recent study has shown that bacterial populations associated to present-day liverworts and mosses include bacteria adapted to aquatic, anaerobic and extreme drought environments, which is consistent with the transition of bryophytes from aquatic to terrestrial conditions (Tang et al., 2016). Due to their key evolutionary position, between unicellular green algae and flowering plants (angiosperms), bryophytes represent interesting organisms to explore the evolution of plant-microbe interactions across the green plant lineage. Recent advances in genome-sequencing projects, including basal angiosperms, gymnosperms, lycophytes, bryophytes, and charophyta algae, have facilitated the identification of gene acquisitions related to terrestrialization, including adaptation mechanisms to interacting microbes. Like other plants, bryophytes have associated microbial communities with beneficial activities. Microbial symbioses appear as one of the possible key innovations in land colonization by plants (Read et al., 2000). The symbiosis with arbuscular mycorrhizal fungi (AMF) and other fungal beneficial associations probably facilitated land colonization by plants improving the capture of nutrients (Delaux et al., 2015). Consistently, plant fossil evidence of fungal structures associated with early bryophytes in 460 and 400 million years old Ordovician sediments has been found (Redecker et al., 2000; Strullu-Derrien et al., 2014). AMF form mutualistic symbiosis with liverwort, improving plant growth and fitness through enhancing nutrient uptake (Humphreys et al., 2010). Liverworts associated with AMF exhibited significantly enhanced photosynthetic carbon gain, increased gametophytic growth and plant fitness, evidenced by greater production of asexual reproductive structures. The benefits of AMF in high CO_2_ atmosphere of the Palaeozoic are amplified by increasing reproduction and growth-limiting rates of phosphorus uptake in a rootless basal land plant (Humphreys et al., 2010). Interestingly, symbiotic signaling genes were already present in algae, and in early land plants diversification of the transcription factors GRAS and half-ABC transporters took place via gene duplication events together with the acquisition of other downstream symbiotic genes (Delaux et al., 2015). The optimization and specificity of symbioses took place in liverworts and angiosperms by independent recruitments of symbiotic nutrient transport systems (Delaux et al., 2015). Rescue assays on flowering plant mutants with orthologs from bryophytes revealed that some plant components required for the establishment of symbiosis are conserved during land plant evolution (Delaux et al., 2013). Cyanobacterial associations with mosses, liverworts, and hornworts have also been reported (DeLuca et al., 2002; Meeks and Elhai, 2002). Interestingly, motile cyanobacterial (*Nostoc* sp.) filaments serving as infection units (hormogonia) require unknown moss signal(s) to move toward and colonize plant tissues (Bay et al., 2013). In the interactions with mosses, cyanobacterial colonization contributes to host nutrition by providing the plant with fixed nitrogen (Bay et al., 2013).

The moss *Physcomitrella patens* (*P. patens*) produces chemical compounds that increase swarming of *Rhizobium leguminosarum*, which is a process of rapid and coordinated movement of bacteria leading to rhizosphere colonization (Tambalo et al., 2014). Movement is probably related to increased expression of genes involved in flagellar filament formation and motility. Bacterial swarming behavior can be influenced by plant strigolactone, since extracts of a strigolactone-deficient moss mutant induce less swarming motility in *R. leguminosarum*. Accordingly, the existence of beneficial interactions between moss and rhizobia, suggest a potential ancient signal perception mechanism between these type of bacteria and plants (Tambalo et al., 2014). Studies of moss-associated bacteria in different ecosystems have shown the presence of diverse microbial populations with a high proportion of antagonistic isolates to fungal pathogens, suggesting that mosses harbor bacteria with antifungal activities that contribute to plant defense (Opelt et al., 2007a,b). Further research is needed to understand the adaptation mechanisms developed by bryophytes and beneficial microorganisms, including chemical communication and the biochemical function of the proteins involved in the establishment of the beneficial interaction.

**FIGURE 1:**
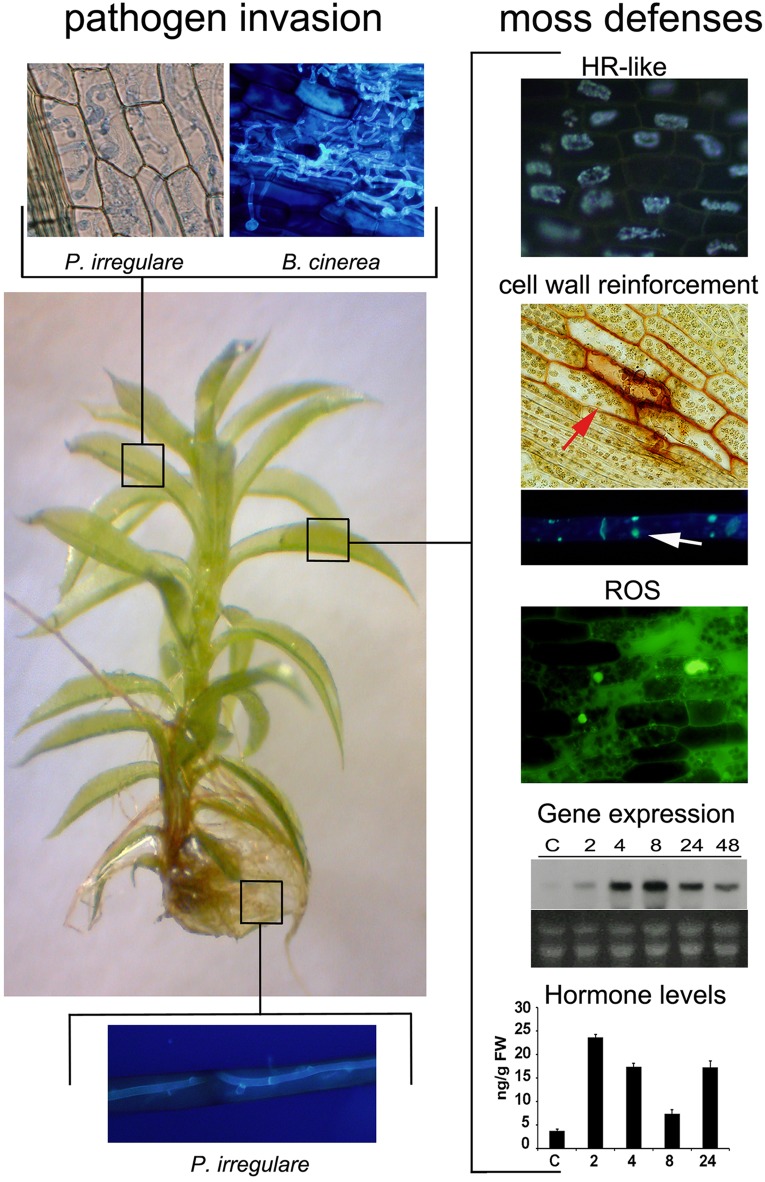
**Pathogen invasion and *P. patens* defense responses.** *P. patens* has one layer of cells in most tissues facilitating microscopic analysis of pathogen colonization processes and moss defense mechanisms (Ponce de León, 2011; Overdijk et al., 2016). Leaves and rhizoids are colonized by different pathogens, which are easily visualized by different staining techniques (leaf; trypan blue and solophenyl flavine, rhizoid: solophenyl flavine) (Ponce de León and Montesano, 2013). Representative pictures of the oomycete *P. irregulare* and the fungus *B. cinerea* pathogen-infected tissues are shown. Plant defenses are activated after pathogen infection, including a HR-like response, cell wall fortification and chloroplast reorientation (red arrow), ROS accumulation, defense gene expression and increase in hormone levels (Ponce de León and Montesano, 2013). Representative pictures of defense responses are shown; HR-response, cell wall fortification (phenolic compounds accumulation, evidenced by stained cell walls) and ROS production in *B. cinerea*-infected leaves, callose deposition in *P.c. carotovorum* elictor treated protonemal cells (white arrow), and expression pattern of the defense gene *Ppalpha-DOX* and auxin levels in response to elicitors of *P.c. carotovorum* at different hours after treatment. The HR-like response was visualized by autofluorescent compounds, cell wall fortification by safranin-O staining, callose deposition by methyl blue staining, and intracellular ROS with 2′,7′-dichlorodihydrofluorescein diacetate. “Graph showing auxin levels as originally published in Alvarez et al. (2016).”

Bryophytes are also colonized by microbial pathogens, including fungi, bacteria, oomycetes and viruses that cause disease evidenced by necrosis and tissue maceration (Ponce de León, 2011). Among bryophytes, *P. patens* has become an attractive model plant for studying plant-pathogen interactions and several moss defense strategies have been described (Ponce de León and Montesano, 2013). The sequenced genome and available genomic resources^1^ (Rensing et al., 2008; Zimmer et al., 2013), allow the identification of genes and metabolic pathways involved in adaptation mechanisms adopted by mosses to defend themselves against microbial pathogens. In addition, targeted knockouts of genes with possible roles in defense can be generated in *P. patens* due to high rates of homologous recombination (Schaefer, 2002). The presence of one layer of cells in most tissues facilitates microscopic analysis of invasion and colonization processes as well as moss defense mechanisms (Ponce de León, 2011; Overdijk et al., 2016; **Figure 1**). This review summarizes the current knowledge on the adaptation mechanisms and the evolution of plant defenses against pathogens, focusing on the latest advances made in the model plant *P. patens*.

## Pathogen Perception and Kinase Activation in *P. Patens*

**FIGURE 2:**
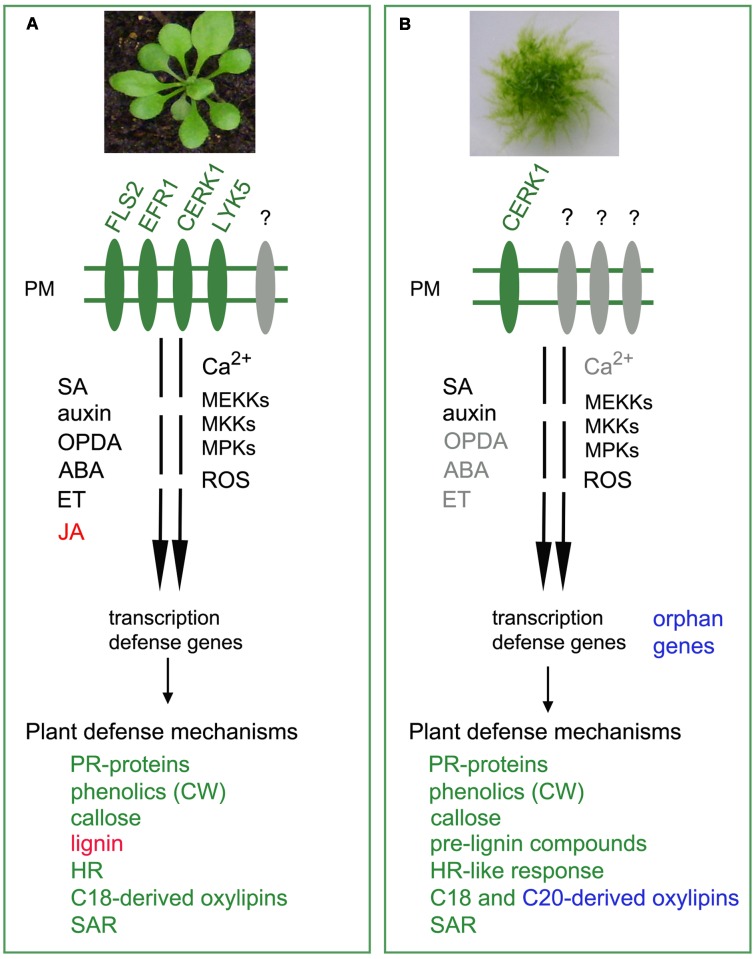
**Activation of defense responses in *Arabidopsis* and *P. patens*.** **(A)** Angiosperms like *Arabidopsis thaliana* sense the presence of pathogen-associated molecular patterns (PAMPs) by plasma membrane (PM) pattern recognition receptors (PRRs), such as FLS2, EFR1, CERK1, and LYK5 (Couto and Zipfel, 2016). Pathogen recognition triggers calcium (Ca^2+^) production and activates a MAPK cascade (Seybold et al., 2014). MAP kinase kinase kinases (MEKKs), MAP kinase kinase (MKKs), and MAP kinase (MPKs) are subsequently activated, leading to production of reactive oxygen species (ROS), and expression of defense genes (Meng and Zhang, 2013). The hormones salicylic acid (SA), jasmonic acid (JA) and its precursor *cis*-oxophytodienoic acid (OPDA), abscisic acid (ABA), auxins, and ethylene (ET) activate the expression of defense genes leading to the activation of defense mechanisms that involves pathogenesis-related (PR) proteins, incorporation of phenolics into the cell wall (CW), deposition of callose and lignin, activation of an hypersensitive response (HR) and systemic acquired resistance (SAR) (Glazebrook, 2005). In response to pathogen infection, oxylipins derived mainly from linoleic acid (18:2) and linolenic acid (18:3; C18 fatty acids) are synthesized (Ponce de León et al., 2015). **(B)** *P. patens* lacks close homologs of the receptors FLS2 and EFR, while a functional CERK1 receptor perceives fungal chitin and bacterial peptidyl glycan (Bressendorff et al., 2016). At least one MEKK, one MKK, and two MPKs (MPK4a and MPK4b) participate in *P. patens* defense responses to fungal chitin (Bressendorff et al., 2016). ROS, SA, and auxin activate expression of defense genes, while JA is not produced (Ponce de León and Montesano, 2013; Reboledo et al., 2015). Further studies are needed to reveal the role of OPDA, ABA and ethylene (names in gray) in moss resistance against pathogens. After pathogen assault, *P. patens* activates several defense mechanisms, including the expression of genes encoding PR proteins, incorporation of phenolics into the CW, callose deposition and accumulation of pre-lignin compounds (Ponce de León and Montesano, 2013; Overdijk et al., 2016). An HR-like response and SAR are also activated in infected mosses, and oxylipins derived from C18 and polyunsaturated C20 fatty acids are synthesized producing a broader range of oxylipins with possible roles in plant defense (Ponce de León et al., 2012, 2015; Winter et al., 2014). Orphan genes, some of them acquired by gene horizontal transfer from fungi and other microorganisms, could also play a role in moss defense against pathogens. Names in red or blue indicate their presence only in traqueophytes or *P. patens*, respectively.

*Physcomitrella patens* is infected by pathogens such as fungi, bacteria and oomycetes leading to tissue damage and disease (Lehtonen et al., 2009; Ponce de León and Montesano, 2013; Overdijk et al., 2016). Several of these pathogens infect mosses in nature, and some of them cause disease in important crops (Ponce de León, 2011). Similarly to angiosperms, *P. patens* perceives the presence of pathogens and activates a defense response evidenced by increased production of reactive oxygen species (ROS), induction of a hypersensitive-like response (HR), cell wall reinforcement, changes in hormones levels and expression of defense genes (Ponce de León et al., 2007, 2012; **Figures 1**, **2**). Angiosperms have pattern recognition receptors (PRRs) at the plasma membrane to detect conserved pathogen-associated molecular patterns (PAMPs), and induce a defense response called PAMP-triggered immunity (PTI), providing protection against non-host pathogens (Jones and Dangl, 2006; Zipfel, 2009). Pathogens adapted to their host plants can deliver effector molecules into plant cells, which target key PTI components and inhibit plant defense (Boller and Felix, 2009; Boller and He, 2009). In turn, plants have a second layer of immune receptors encoded by resistance (*R*) genes to detect directly or indirectly the effector proteins leading to effector-triggered immunity (ETI), which is highly specific and often accompanied by a HR and systemic acquired resistance (SAR). Up to date, several PAMPs and the corresponding PRRs have been identified in angiosperms. In *Arabidopsis thaliana* (*A. thaliana*), the receptors FLS2 (for flagellin-sensing 2), EFR1 (for Elongation factor tu receptor 1), and LYK1/CERK1 (for LysM-containing receptor-like kinase1/chitin elicitor receptor kinase1), recognize bacterial flagellin, the elongation factor Tu, and the fungal chitin, respectively (Gómez-Gómez and Boller, 2000; Zipfel et al., 2006; Miya et al., 2007). LYK4 and LYK5 are also important for chitin signaling and immunity in *A. thaliana* (Wan et al., 2012; Cao et al., 2014). LYK5 was identified as the receptor that recognizes chitin in *A. thaliana*, forming a chitin inducible complex with CERK1 to induce plant immunity (Cao et al., 2014). Interestingly, *P. patens* lacks close homologs of the receptors FLS2 and EFR (Boller and Felix, 2009), which is in accordance to the insensibility of moss cells to flagellin (flg22) and the elongation factor Tu (Bressendorff et al., 2016). A functional CERK1 receptor was recently identified in *P. patens* and PAMPs such as fungal chitin and bacterial peptidyl glycan are perceived by this receptor (Bressendorff et al., 2016; **Figure 2**). Mutation in moss PpCERK1 leads to reduced defenses, including less expression of defense genes and less incorporation of phenolic compounds associated to cell wall defenses, showing that PTI is an ancient plant defense response (Bressendorff et al., 2016). Further studies are needed to understand chitin perception by *P. patens*, including analyses of molecular complexes related to PpCERK1 and putative LYK5-like receptors leading to the activation of moss immunity.

Other receptor-like kinases (RLKs) such as *Catharanthus roseus* RLK1-like (CrRLK1L) proteins are also linked to plant-pathogen interactions, and orthologs have been found in *P. patens*, the liverwort *Marchantia polymorpha* (*M. polymorpha*) and the primitive vascular plant *Selaginella moellendorffii* (*S. moellendorffii*) (Galindo-Trigo et al., 2016). In *A. thaliana* two CrRLK1Ls have been directly associated with defense responses to biotic stress, and expression data in elicitor-treated and pathogen-infected tissues suggest that additional CrRLK1Ls are involved (Lindner et al., 2012; Nissen et al., 2016). In *A. thaliana*, one of these CrRLK1Ls (Feronia) is phosphorylated upon treatment with flg22, and it is speculated that it may act as a co-receptor of FLS2 (Keinath et al., 2010). CrRLK1Ls could have a conserved role throughout the plant lineage recognizing cell wall changes caused by invading fungal hyphae, and relocating proteins involved in resistance toward the penetration site, such as MLO (Mildew Resistance Locus O) (Lindner et al., 2012). Interestingly, a closely related CpRLK1 protein was identified in a charophycean unicellular alga and functional characterization suggests that this protein could also be involved in cell wall stability sensing, specifically by detecting cell wall integrity during sexual reproduction (Galindo-Trigo et al., 2016). Other plant RLKs related to defenses to biotic stress are the RLK/Pelle genes, which expanded significantly with the establishment of land plants, suggesting an adaptation mechanism to fast-evolving pathogens (Lehti-Shiu et al., 2009). Consistently, while *P. patens* and traqueohpytes have large RLK/Pelle gene families, only two predicted members with no extracellular domain were identified in the alga *Chlamydomonas reinhardtii* (Lehti-Shiu et al., 2009). *P. patens* has also several putative RLKs with high similarities to the rice receptor OsXa21 (*Oryza sativa Xanthomonas* resistance 21) as well as homologous proteins to the corresponding interacting proteins (PpXB3s) (Tanigaki et al., 2014). Some of these PpXB3s interacts with several PpRLKs in a yeast two hybrid system, suggesting that *P. patens* could have an OsXa21-like RLK-type sensing system to detect molecules from pathogens (Tanigaki et al., 2014).

Several putative intracellular receptor genes (R genes) have been identified in *P. patens*, including kinase-Nucleotide Binding Site (NBS)-Leucine Rich Repeat (LRR) receptors and Toll/interleukin-1 like Receptor (TIR)-NBS-LRR (Akita and Valkonen, 2002; Xue et al., 2012; Tanigaki et al., 2014). Since algae do not have NBS-LRR, TIR-NBS-LRR, or TIR-LRR homologs (Sarris et al., 2016), R-like genes appeared in basal land plants probably as an adaptation mechanism to detect pathogens and activate a defense response. The NBS-LRR containing protein family has expanded in flowering plant species and this increase is associated with polyploidy or ancient polyploidization events. Surprisingly, the repertoire of NBS-LRR and other putative R-genes is much smaller in the primitive vascular plant *S. moellendorffii* compared to *P. patens* (Sarris et al., 2016). Further studies are needed to understand if pathogen effectors could suppress moss defense responses, and whether they could be detected directly or indirectly by some of these R-like proteins activating an ETI in *P. patens*.

Calcium (Ca^2+^) is a second messenger essential in both angiosperm PTI and ETI responses leading to ROS and nitric oxide production, and induction of plant defense genes (Zhang et al., 2014). An increase of cytosolic Ca^2+^ is generated after pathogen perception, and Ca^2+^-dependent protein kinase (CDPK) and MAPK pathways modulate plant biotic stress signaling (Seybold et al., 2014). To our knowledge the role of Ca^2+^ and CDPKs in moss-pathogen interactions has not been studied so far. MAPK cascades are involved in signaling multiple defense responses, including the biosynthesis and signaling of defense hormones, ROS generation, defense gene activation, phytoalexin biosynthesis, cell wall reinforcement, and HR cell death (Meng and Zhang, 2013). MAPK cascade typically consists of a MAP kinase kinase kinase (MEKK), a MAP kinase kinase (MKK), and a MPK (Rodriguez et al., 2010). In *A. thaliana* at least four MPKs (MPK3/4/6 and 11) are activated by PRRs (Fiil et al., 2009; Bethke et al., 2012). Activation of MPK4 requires MEKK1 and MKK1/MKK2 (Gao et al., 2008). It was recently discovered that in *P. patens* at least two MPKs are rapidly phosphorylated and activated in response to bacterial and fungal PAMPs (Bressendorff et al., 2016). *P. patens* MKK1b, MKK1c, MPK4a, and MPK4b transcript levels are rapidly induced after chitin treatment. By generating targeted knockout mutants in several kinases it was shown that at least one MEKK, one MKK, and two MPKs (MPK4a and MPK4b) are components of an immune pathway in *P. patens*, and are required for defense responses to the fungal chitin (Bressendorff et al., 2016). In addition, MPK4a mutants result in loss of basal resistance to pathogens, evidenced by reduced cell wall associated defenses and reduced expression of defense genes, showing that a basal defense signaling pathway is already present in *P. patens*. In contrast to *A. thaliana*, abiotic stress in *P. patens* does not activate MPK4a or any other MPK, suggesting that MPK4a is specific to immunity in basal land plants like mosses (Bressendorff et al., 2016).

## Reactive Oxygen Species and Hypersensitive Response Cell Death in P. Patens Defense

After pathogen recognition, one of the first plant responses involves the production of ROS, which are directly toxic to pathogens and are involved in cell wall reinforcement, HR induction and signaling leading to gene expression (Torres et al., 2006). Several enzymes are involved in ROS production during plant-pathogen interactions, including peroxidases and NADPH oxidases (Torres et al., 2002; Bindschedler et al., 2006). Like in angiosperms, several lines of evidence suggest that ROS participate in *P. patens* defense against pathogens. Exposure of *P. patens* tissues to the fungal elicitor chitosan leads to rapid production of ROS, increased expression of alternative oxidase (PpAOX), NADPH-oxidase (PpNOX), and increased peroxidase (PpPrx34) activity (Lehtonen et al., 2009, 2012a). Rapid ROS production was also evidenced in single moss leaf cells in contact with *B. cinerea* hyphae (Ponce de León et al., 2012). Other proteins related to oxidative stress such as cysteine peroxiredoxin, several thioredoxins, and copper/zinc superoxide dismutases are secreted to the apoplast after chitosan treatment (Lehtonen et al., 2014). The expression of a gene encoding a PpTSPO1 protein involved in mitochondrial tetrapyrrole transport is also induced by chitosan (Lehtonen et al., 2012a). Peroxidases, thioredoxins, and glutathione *S*-transferases transcript levels increase after treatment with bacterial culture filtrate of *Pectobacterium carotovorum* subsp. *carotovorum* (*P.c. carotovorum*) containing elicitors such as harpin and plant cell wall degrading enzymes secreted by this bacteria (Alvarez et al., 2016). The liverwort *Dumortiera hirsute* produces superoxide radicals by the action of apoplastic peroxidases and tyrosinases leading to an extracellular oxidative burst during rehydration (Li et al., 2010). Liverwort peroxidases may also protect plant tissues against pathogens by producing ROS and melanin-like compounds (Li et al., 2010). Functional analyses in *P. patens* knockouts have shown that PpPrx34 and PpTSPO1 are important for resistance against pathogens (Lehtonen et al., 2009, 2012a), indicating that ROS production is a common defense mechanisms in mosses and angiosperms.

Chloroplasts are a rich source of ROS and these organelles are repositioned in *P. patens* cells toward the penetration site of several pathogens (Oliver et al., 2009; Lehtonen et al., 2012b; Reboledo et al., 2015; **Figure 1**). Although the precise role of chloroplast repositioning is at present unknown, plastidic ROS sources as well as the production of specific chloroplastic molecules could be part of the defense response to biotic stress. Interestingly, *Nicotiana benthamiana* chloroplasts send out dynamic tubular extensions called stromules during plant defense responses, which form connections with the nucleus (Caplan et al., 2015). These stromules transport pro-defense signals such as H_2_O_2_ into the nucleus during plant defense. Like in angiosperms, *P. patens* induces accumulation of cytoplasm and repositioning of the nucleus to pathogen contact sites, and rearrangement of the actin cytoskeleton after *Phytophthora capsici* (*P. capsici*) infection (Overdijk et al., 2016). Actin cytoskeleton is important for stromules formation and probably facilitates vesicle delivery to the infection site (Takemoto et al., 2006; Caplan et al., 2015). Further studies are needed to understand the role of chloroplasts repositioning during pathogen infection, including the relation with the pathogen penetration site, nucleus movement and cytoskeleton rearrangement.

The HR response contributes to plant resistance to biotrophic pathogens by confining the pathogen and limiting its growth, while necrotrophic pathogens actively stimulate the HR to enhance tissues colonization (Govrin and Levine, 2000; Jones and Dangl, 2006). *P. patens* cells infected or treated with elicitors of HR-inducing pathogens such as *Botrytis cinerea* (*B. cinerea*), *Colletotrichum gloeosporioides* (*C. gloeosporioides)*, and *P.c. carotovorum*, induce a HR-like reaction (Ponce de León et al., 2007; Reboledo et al., 2015). Typical programmed cell death (PCD) hallmarks are present in elicitor-treated or pathogen-infected moss tissues, including chloroplasts breakdown, accumulation of autofluorescent compounds, cytoplasmic shrinkage, fragmentations of the nucleus and nuclease activities (Ponce de León et al., 2007, 2012; Lawton and Saidasan, 2009).

In plants and algae, ROS generated during pathogen infection induce autophagy, which is a regulated process involved in recycling intracellular components (Pérez-Pérez et al., 2012). Autophagy is important in plant innate immunity and is involved in HR cell death (Lenz et al., 2011). *A. thaliana* autophagy deficient mutants (*atg*) develop spreading necrosis upon *Alternaria brassicicola* infection, and fungal toxin fumonisin B1 treatment (Lenz et al., 2011). However, *atg* mutant plants are more resistant to *Pseudomonas syringae* pv. *tomato* infection, suggesting that different mechanisms related to autophagy contribute to resistance after infection with different pathogens (Lenz et al., 2011). Like in *A. thaliana*, *P. patens atg5* mutant shows early senescence under nutrient deficient conditions, which was partly explained by amino acid imbalance due to lack of cytoplasmic degradation by autophagy (Mukae et al., 2015). Although further studies are needed to understand the involvement of autophagy in moss defense against pathogens, preliminary results suggest that *atg5 P. patens* mutant plants are more susceptible than wild type plants to *Sordariomycetes* sp. fungus infection (Bressendorff, 2012).

## Functional Evolution of Hormonal Pathways and Their Role in Plant Defense

The plant hormones salicylic acid (SA), jasmonic acid (JA), and ethylene have a central role in defense against pathogens in angiosperm. Additionally, other hormones such as abscisic acid (ABA), auxins, cytokinins, gibberellins, and brassinosteroids have emerged as regulators in plant immunity (Denancé et al., 2013). These hormones function in complex networks to regulate many aspects of plant resistance. In bryophytes, only few studies have related some of these hormones with defense against pathogens. However, genome-wide analyses of multiple species have been useful to gain insights into the origin and evolution of hormonal signaling components across the plant lineage. While algal genomes have the orthologs of only some components of the SA, JA, ABA signaling pathways, land plants have all the components, suggesting these signaling pathways might have developed during land colonization by plants (Wang et al., 2015). Orthologs of the Non-expresser of Pathogenesis-Related (NPR) genes have been found in *P. patens* (Wang et al., 2015). Studies in *A. thaliana* have identified NPR3 and NPR4 as SA receptors, while NPR1 is a key regulator in SAR, which is an important long distance signaling mechanism present in flowering plants that provides broad spectrum and long-lasting resistance to secondary infections throughout the plant (Fu et al., 2012). NPR3 and NPR4 are negative regulators of immunity, acting as substrate adaptors for the recruitment of NPR1 to an E3-ubiquitin ligase, which leads to NPR1 degradation by the proteasome. When NPR1 is not degraded and accumulates in the cytoplasm, it activates transcription factors such as WRKYs and TGAs, turning on defense genes encoding pathogenesis related (PR) proteins such as PR-1, PR-2, and PR-5 (Fu et al., 2012). SA levels increase in *P. patens* after *B. cinerea* infection and treatments with SA induce the expression of the defense gene phenylalanine ammonia lyase (*PAL.1*), suggesting that SA is involved in moss defense (Ponce de León et al., 2012). Consistently, SA application to *P. patens* colonies increases resistance to *P. wasabiae* (Andersson et al., 2005). Furthermore, the moss *Amblystegium serpens* activates a SAR-like response upon inoculation with *Pythium irregulare* (*P. irregulare*) or treatment with β-1,3 glucan increasing resistance against subsequent *P. irregulare* infections (Winter et al., 2014). This finding suggests that a SAR-like response exists before the divergence of mosses and vascular plants. Further research is needed to identify if SA and/or other signaling molecules participate in moss systemic responses. The use of SA-deficient transgenic moss plants, overexpressing a bacterial salicylate hydroxylase (encoded by NahG) that degrades SA, will help to elucidate if SA is involved in local and systemic moss defense.

Upon pathogen attack, JA-isoleucine accumulates rapidly in angiosperms promoting the interaction between the receptor COI1 and the repressor proteins JAZ, leading to the ubiquitination and subsequent degradation of JAZs through the proteasome (Chini et al., 2007). The degradation of JAZ relieves the repression on MYC transcription factors allowing the expression of JA-responsive genes. *P. patens* encode the orthologs of all JA signaling components, although moss and liverworts do not produce the oxygenated fatty acid (oxylipin) JA (Ponce de León et al., 2015; Yamamoto et al., 2015). The precursor 12-oxo-phytodienoic acid (OPDA) accumulates in *P. patens* tissues infected with *P. irregulare*, *P. debaryanum* (*P. debaryanum*) and *B. cinerea* (Oliver et al., 2009; Ponce de León et al., 2012). Similarly as in *A. thaliana*, OPDA could act as a signaling molecule in bryophytes leading to the induction of defense related genes (Taki et al., 2005). Surprisingly, *P. patens* and *A. thaliana* respond similarly to OPDA or JA, showing reduced rhizoid length and moss colony size, and reduced growth of seedlings and roots, respectively (Ponce de León et al., 2015). Furthermore, jasmonates (JA and methyl jasmonate), and OPDA induce the expression of *PAL.2*, suggesting that all components needed for sensing JA and transducing the signal are present in basal land plants (Oliver et al., 2009). Since genes encoding proteins homologous to the JA-isoleucine receptor COI1 are present in the *P. patens* genome, other oxylipins than JA-isoleucine could be detected resulting in the activation of a defense response (Ponce de León et al., 2012). Interestingly, the binding sites are well conserved between COI orthologs of *P. patens*, the liverwort *M. polymorpha* and *A. thaliana* COI1 (Wang et al., 2015). The similarities between COI1 and the auxin receptor TIR1, at the amino acid sequence level and their mode of actions, as well as the fact that they probably originated from gene duplication events in bryophytes, suggest that JA and auxin pathways may have evolved in parallel from a common ancestor that later diverged by successive mutations to bind different hormones (Chico et al., 2008; Wang et al., 2010). Jasmonates could have evolved later in plant evolution as a result of selection pressures by insects and novel pathogens (Groen and Whiteman, 2014).

Abscisic acid plays a role in priming of callose biosynthesis after pathogen recognition and it induces the expression of defense genes in traqueophytes (Adie et al., 2007). While several studies have demonstrated the involvement of ABA in moss defense against abiotic stress (Cuming and Stevenson, 2015), little is known so far about the role played by ABA in defense against biotic stress. *P. patens* plants infected with *B. cinerea* show a small increase of ABA, however, it cannot be discarded that it is produced by the pathogen (Ponce de León et al., 2012). In *P. patens*, ABA induces the synthesis of defense proteins such as a RPM1-like R protein, a lipoxygenase (LOX) involved in the production of oxylipins, an intracellular pathogenesis-related protein, a *N*-hydroxycinnamoyl/benzoyl transferase involved in the production of phytoalexin, a hydroxyproline-rich glycoprotein and a proline-rich protein involved in cell wall reinforcement, an ascorbate peroxidase and a peroxiredoxin, suggesting that ABA could be involved in moss defense against pathogens (Wang et al., 2010).

Bryophytes produce ethylene and the orthologs of the ethylene sensing and signaling components are present in several land plant genomes (Wang et al., 2015). *P. patens* has two putative genes encoding enzymes involved in the synthesis of the ethylene precursor, 1-aminocyclopropane 1 carboxylic acid (ACC) (Rensing et al., 2008), although their activities need to be confirmed. At least one of the seven ETR-like ethylene receptors binds ethylene in *P. patens* and this receptor is needed for a full ethylene water submergence moss response (Yasumura et al., 2012), suggesting that ethylene signaling was already present in mosses (Wang et al., 2015). Until present, there are only few lines of evidence suggesting that ethylene participates in moss defenses against pathogens. In *P. patens*, chitosan treatment and *P. capsici* infection induce the expression of the ethylene responsive transcription factor ERF2 and ERF5, respectively (Bressendorff et al., 2016; Overdijk et al., 2016), and treatment with ACC induces the expression of moss defense genes (Ponce de León and Montesano, 2013). In addition, several putative *P. patens* AP2/ERF encoding transcripts increase after bacterial elicitor treatment (Alvarez et al., 2016). Similarly, increased production of ethylene in *A. thaliana* leads to a cascade of transcription factors, including downstream ERF-like transcription factors that control the expression of several defense genes (Broekaert et al., 2006). Further studies using moss mutants in key components of the ethylene pathway, including ETR-like receptors will help to elucidate their role in defense against pathogens.

Auxin is produced in algae, and charophyte genomes have several components of auxin signaling and transport machinery, including ARFs, AUX/IAAs, and PINs, suggesting that AUX signaling originated in algae (Wang et al., 2015). In *P. patens* and *M. polymorpha* transcriptomes orthologs of all the signaling and transport core components were found, except for the liverwort Auxin-Binding Protein1, probably due to low coverage of the transcriptome (Wang et al., 2015). In *P. patens*, auxin levels increase in *P.c. carotovorum* elicitor-treated and *Pythium*-infected tissues (Mittag et al., 2015; Alvarez et al., 2016). Furthermore, the use of a moss reporter line harboring the auxin inducible promoter GH3 (indole-3-acetic acid amino acid conjugate synthetases) fused to β-glucuronidase, shows that auxin signaling is induced in response to *P.c. carotovorum* elicitors and the pathogens *P. irregulare*, *P. debaryanum*, and *C. gloeosporoides* (Mittag et al., 2015; Reboledo et al., 2015; Alvarez et al., 2016). Like in *A. thaliana* where auxin induces the expression of genes and activates defense, auxin has been associated to *P. patens* defense against pathogens (Mittag et al., 2015). High auxin levels present in *P. patens* GH3 double knockout plants increase resistance against *P. debaryanum* (Mittag et al., 2015), and auxin induces the expression of the defense gene *PAL.1* (Reboledo et al., 2015).

Orthologs of all the key cytokinin signaling components were identified in the charophyte and land plants genomes, suggesting that this hormonal signaling pathway originated in algae (Wang et al., 2015). For brassinosteroids, several of the signaling transduction components are present in algal genomes, although the key regulator BKI1 (Brassinosteroid Insensitive1Kinase Inhibitor) is found in angiosperms and not in algae, bryophytes, and lycophytes, suggesting that the brassinosteroids signaling pathway originated before the emergence of angiosperms (Wang et al., 2015). The roles played by cytokinin and brassinosteroids in moss defense have not been studied so far. Apparently, *P. patens* does not produce gibberellin, although it has part of the gibberellin biosynthetic pathway from geranylgeranyl diphosphate to *ent*-kaurenoic acid via *ent*-kaurene (Miyazaki et al., 2015). In angiosperms, the gibberellin signal is perceived and transduced by the GID1 gibberellin receptor/DELLA repressor pathway. A comparative analysis of gibberellin signaling components has shown that land plant genomes, except *M. polymorpha* probably due to low coverage of the transcriptome, encode the orthologs of all the core components of gibberellin signaling (Wang et al., 2015). While GID1 and DELLA do not interact with each other in *P. patens*, the lycophyte *S. moellendorffii* produces gibberellin and has functional GID1-DELLA signaling components (Yasumura et al., 2007). These results suggest that gibberellin signaling originated after the evolutionary split of bryophytes and traqueophytes. In contrast to flowering plants, *P. patens* has a bifunctional *ent*-kaurene synthase (KS) which has both *ent*-diphosphate synthase and *ent*-KS activities (Hayashi et al., 2006). *P. patens* mutant in this bifunctional CPS/KS has a phenotype with defective development that is restored with *ent*-kaurene or *ent*-kaurenoic acid (Miyazaki et al., 2015). The homologous diterpene cyclase (HpDTC1) from the moss *Hypnum plumaeforme* (*H. plumaeforme*) catalyzes a two-step cyclization reaction of geranylgeranyl diphosphate to *syn*-pimara-7,15-diene, which is a precursor for phytoalexin momilactones (Okada et al., 2016). HpDTC1 gene expression and momilactone accumulation is induced by chitosan and *B. cinerea* infection, suggesting a defensive role for momilactone biosynthesis in this moss (Okada et al., 2016). Interestingly, *cis*-OPDA but not JA enhanced HpDTC1 expression and momilactone accumulation, indicating that this oxylipin could regulate momilactone biosynthesis in *H. plumaeforme* (Okada et al., 2016). The tetracyclic diterpene 16α-hydroxykaurane (Von Schwartzenberg et al., 2004), is produced in *P. patens* tissues treated with chitosan, and transcripts levels encoding enzymes associated to kaurenes production increase, suggesting that this type of metabolites are also involved in moss defense (Lawton and Saidasan, 2009*).* Taken together, some of these hormonal signaling pathways represent innovations that probably played a role during the adaptation of terrestrial plants to microbial pathogens.

## Phenylpropanoid and Oxylipins Pathways in Moss Adaptation to Biotic Stress

Genome analysis has revealed that *P. patens* has large gene families involved in protection against stresses (Rensing et al., 2008). Examples are the expansion of heat shock proteins 70, photoprotective early light-induced proteins, and enzymes involved in the phenylpropanoid pathway such as PALs and chalcone synthases (CHSs) (Lang et al., 2005; Rensing et al., 2008; Wolf et al., 2010). Interestingly, metabolic genes have been maintained in excess in *P. patens* following genome duplication, which took place about 45 million years ago, and this could be related to a more generalist metabolic gene complement that allow mosses to grow in more diverse habitats (Rensing et al., 2007). In addition, plant colonization required improved defense mechanisms to biotic challengers, including the production of novel or more specialized secondary metabolites. The phenylpropanoid pathway is probably one of the most relevant adaptation mechanisms that have evolved in early land plants to cope with abiotic stresses and to avoid invasion by already diversified microbial soil communities (Emiliani et al., 2009). In *P. patens*, the phenylpropanoid pathway is activated in response to *B. cinerea*, *C. gloeosporioides*, two *Pythium* species, *P. capsici*, chitin, and secreted elicitors of the pathogenic bacteria *P.c. carotovorum* (Ponce de León et al., 2007, 2012; Oliver et al., 2009; Reboledo et al., 2015; Alvarez et al., 2016; Bressendorff et al., 2016; Overdijk et al., 2016). Several genes from the shikimate and phenylpropanoid pathways are induced after *P.c. carotovorum* elicitor treatment, including genes encoding a chorismate synthase, a chorismate mutase, PALs, a cinnamic acid 4-hydroxylase, a 4-coumarate CoA Ligase, CHSs, CHIs, a flavanone 3-hydroxylase and a cinnamyl alcohol dehydrogenase (Alvarez et al., 2016). The phenylpropanoid metabolism generates in angiosperms an enormous array of secondary metabolites with different roles in defense against pathogens, such as SA, flavonoids, isoflavonoids, phytoalexins, and lignins (Dixon and Paiva, 1995). Metabolites from the phenylpropanoid pathway have also been identified in *P. patens*, including caffeic acid, the cinnamate conjugate, caffeoylquinic acid, and the intermediate of lignin biosynthesis, chlorogenic acid (Erxleben et al., 2012). Cinnamic acid increases in *P.c. carotovorum* elicitor-treated moss tissues and genes encoding Dirigent proteins involved in monolignol coupling are also induced by these elicitors as well as by several pathogens (Ponce de León et al., 2012; Reboledo et al., 2015; Alvarez et al., 2016). This suggests that cinnamic acid-derived and monolignol-containing compounds are produced in *P. patens* after pathogen assault. The presence of lignin in bryophytes is still controversial and *P. patens* could use cell wall-bound phenolics that resemble lignin for defensive purposes. Lignin-like polymers have also been identified in cell walls of algae, other mosses, liverworts and hornworts where they are proposed to principally serve as a defense mechanism against microorganisms and UV radiation (Ligrone et al., 2008). Callose is a high-molecular weight β-(1,3)-glucan polymer involved in plant cell wall defenses and it is usually associated to phenolic compounds, polysaccharides and antimicrobial proteins forming cell wall appositions (papilla) (Luna et al., 2011). Cell wall appositions were identified in *P. patens* tissues inoculated with *P. debaryanum*, *P. capsici*, *P. infestans* (*P. infestans*) and *Apiospora montagnei* (Oliver et al., 2009; Lehtonen et al., 2012b; Overdijk et al., 2016). Similarly, papilla formation was observed in the moss *Funaria hygrometrica* inoculated with the fungus *Atradidymella muscivora* and *Coniochaeta velutina* (Davey et al., 2009, 2010). Papilla-like structures are capable of blocking *P. infestans* and *P. capsici* penetration and further colonization, which are defensive barriers also observed in angiosperms (Overdijk et al., 2016).

*Physcomitrella patens* has two CHIs, which are enhancer of flavonoid production genes encoding type IV CHI proteins with no CHI activity (Ngaki et al., 2012), and the conversion of flavanone to flavone by flavone synthase seems to be absent in mosses (Koduri et al., 2010). Both *CHI* genes are induced in response to *P.c. carotovorum* elicitor treatment (Alvarez et al., 2016), suggesting that they participate in moss defense. Since flavonoids are important defense compounds in plant responses to pathogens and insects, further analyses are needed to characterize enzymatic activities of the pathway and to have a broader metabolic scenario in *P. patens* tissues under biotic stress conditions.

*Physcomitrella patens* has a high proportion of proteins involved in metabolism, and alternative metabolic pathways have been discovered in this moss (Lang et al., 2005). Interestingly, *P. patens* has a lipoxygenase with a hydroperoxidase and fatty acid chain-cleaving lyase activity, which uses C18-fatty acids (linolenic and linoleic acid) and C20-fatty acids (20:4 arachidonic and 20:5 eicosapentaenoic acids) as substrates yielding a broader range of oxylipins than angiosperms (**Figure 2**; Senger et al., 2005; Wichard et al., 2005; Anterola et al., 2009). The high abundance of long and very long chain fatty acids as well as the presence of oxylipins derived from C20:4 and C20:5 represent a metabolic difference between mosses and angiosperms that may provide a metabolic advantage to adapt to abiotic stress (Mikami and Hartmann, 2004), and probably to protect themselves against pathogen invasion. Mosses could have evolved the ability to produce C20-derived oxylipins for defensive purposes against pathogens, although further research is needed to determine if some of them have antimicrobial activities or induce defense genes. Like in angiosperms, pathways leading to oxylipin biosynthesis are activated in *P. patens* after pathogen assault or elicitor treatment, evidenced by increased endogenous levels of free unsaturated fatty acid and induced expression of several lipases, LOXs, allene oxide synthases, OPDA reductases and alpha-Dioxygenases (alpha-DOX) (Lehtonen et al., 2014; Ponce de León et al., 2015; Alvarez et al., 2016; Bressendorff et al., 2016). These evidences show that different LOXs and alpha-DOX pathways are activated after pathogen assault, leading to the production of different oxylipins that could play different roles in moss defense. Interestingly, LOX-derived oxylipins, synthesized from C20 and C18 polyunsaturated fatty acids, are also produced in algae, where they function in defense against an algal pathogen (Bouarab et al., 2004). Oxilipin profiling after pathogen assault and the generation of multiple knockouts plants in the different enzymes of these pathways will help to elucidate their role in *P. patens* defense. Like in angiosperms, moss tissues treated with pathogens increase production of oxylipins derived from the alpha-DOX, which reduce cellular damage caused by elicitors of *P.c. carotovorum* (Machado et al., 2015).

## Moss Defense Genes and Gene Acquisition By Horizontal Transfer

Different genes encode proteins that are involved in plant resistance against pathogens, and generally their expression is up-regulated when plants are attacked by bacteria, fungi, oomycetes, or viruses. In angiosperms, the expression of genes encoding PR proteins increases at the infection site and during SAR (van Loon et al., 2006). Several PR encoding genes are induced in *P. patens* after *P.c. carotovorum* elicitor treatment or pathogen infection, including PR-1, PR-10 and PR-3 (chitinase) (Ponce de León et al., 2007; Oliver et al., 2009; Alvarez et al., 2016). Moreover, thaumatin-like protein (PR-5) and chitinases are secreted to the extracellular space when moss plants grown in liquid medium are treated with a fungal elicitor (Lehtonen et al., 2014). Transcript levels of genes encoding transcription factors, betaine-aldehyde dehydrogenase and proteins involved in modification or reinforcement of the cell wall such as Dirigent proteins and xyloglucan:xyloglucosyl transferases also increase in moss tissues treated with bacterial secreted elicitors (Alvarez et al., 2016).It was recently shown that chitin oligosaccharides induce the expression of *P. patens* chitinase, which inhibits the growth of the fungus *Trichoderma viride*, suggesting that this enzyme could play a role in moss defense (Kobaru et al., 2016). Moss lines overexpressing PpPR-10 are more resistant to *P. irregulare* infection and accumulate unknown cell wall polymers, probably reinforcing the cell walls and increasing resistance to pathogens (Castro et al., 2016). PpPR-10 binds cytokinin (Gonneau et al., 2001), however, the biological relevance of this binding capacity in plant defense against pathogens is currently unknown. Co-expression of PpPR-10 with moss genes encoding enzymes of the phenylpropanoid pathway, together with the fact that some PR-10 proteins from other plants are related to phenylpropanoids biosynthesis, suggest that PpPR-10 could also affect phenolic compounds repertoire in moss tissues (Castro et al., 2016). Other proteins with possible roles in defense are also secreted to the apoplast in chitosan-treated moss tissues, including a D-mannose binding lectin protein (Lehtonen et al., 2014), which could act by recognizing pathogen surfaces and activating a moss defense response similarly as in angiosperms (Hwang and Hwang, 2011).

Several genes present in *P. patens* and associated to defense were acquired by horizontal gene transfer, providing an adaptive advantage that probably played a role in defense against microbial colonization in basal land plants. Examples include the bifunctional CPS/KS enzyme, which probably came from fungi by horizontal gene transfer, and unifunctional CPS and KS evolved gradually in angiosperms (Mukherjee, 2015). PAL was probably transferred horizontally from soil bacteria (Emiliani et al., 2009), and the major intrinsic protein acquired from bacteria probably facilitates the transport of glycerol in *P. patens* (Gustavsson et al., 2005). The acquisition of glycerol transporters in plants could have been important for symbiotic fungi and bacteria, representing an effective mechanism for transferring carbon sources from the plant to microbes (Wei et al., 2004; Gustavsson et al., 2005). Several genes acquired in *P. patens* by horizontal transfer from bacteria and fungi are directly or indirectly related to plant defense responses and stress tolerance. A glutamate-cysteine ligase acquired from bacteria is involved in glutathione formation, which is important for plant disease resistance (Yue et al., 2012). Other acquired genes encoding proteins with possible roles in pathogen resistance include a killer toxin Protein, a bifunctional nuclease, and an acid phosphatase (Yue et al., 2012). These acquired genes could have expanded the metabolic capabilities of *P. patens* to better adapt to pathogen infection as well as to beneficial microbes. *P. patens* has 13% orphan genes (Zimmer et al., 2013), and some of them could represent innovative adaptive strategies to biotic stress. Consistently, some of these orphan genes were identified in a library of *P.c. carotovorum* elicitor-treated *P. patens* tissues (Alvarez et al., 2016), suggesting that they could play a role in defense against pathogens. Due to the high number of orphan genes expressed in *P. patens* during abiotic stresses, species- or lineage-specific genes could have played an important role in the acquisition of abiotic stress tolerance in mosses (Khraiwesh et al., 2015). Further studies during biotic stress will help to elucidate if orphan genes could also play a role in moss resistance against pathogens.

## Conclusion

As early land-growing plants, bryophytes like *P. patens* adapted cellular and molecular responses to interact with beneficial microbes and to cope with microbial pathogens during their life cycle. Comparative studies between beneficial and pathogenic interactions open the possibility to gain insights into the molecular mechanisms involved during coevolution, and how basal land plants have evolved the ability to distinguish between these different types of microbes. Further research is needed to identify and characterize more moss PRRs and possible targets of pathogen effector proteins that inhibit PTI or ETI mediated signaling in bryophytes. Studies conducted so far have shown that several defense mechanisms against pathogen infection are conserved among moss and angiosperms. Some of these mechanisms emerged when plants colonized land, probably influencing fitness and conferring adaptive advantages to biotic stressors. Certain precursor stages of defense mechanisms were already present in the algal ancestor of plants, while several moss genes associated to defense were acquired with terrestrialization, either via horizontal gene transfer from microbes or by duplication events and probably further diversification and functional specialization. The expansion of metabolic gene families, the production of novel metabolites, and the presence of orphan genes with possible roles in *P. patens* defense, suggest that 470 million years of divergent evolution lead to the development of alternative defense strategies for survival in extant moss and angiosperms. The efficient transient transformation and generation of *P. patens* knockouts in several genes using CRISPR-Cas9 open new perspectives for functional analysis of gene families that participate in moss defense (Lopez-Obando et al., 2016). Furthermore, genome wide expression analysis, generation of knockout moss plants, and comparative studies with species representing different plant lineages, will provide new insights into the evolutionary dynamics, diversity and origin of genes, including moss-specific genes, involved in defense against biotic stressors and adaptation mechanisms that played a crucial role in colonization by land plant ancestors.

## Author Contributions

IPDL conceived the review and wrote the manuscript. MM participated in the discussions and helped to draft and write the manuscript.

## Conflict of Interest Statement

The authors declare that the research was conducted in the absence of any commercial or financial relationships that could be construed as a potential conflict of interest.

The reviewer EO and handling Editor declared their shared affiliation, and the handling Editor states that the process nevertheless met the standards of a fair and objective review.

## Footnotes

1 https://phytozome.jgi.doe.gov

## References

[B1] Adie B. A. Pérez-Pérez J. Pérez-Pérez M. M. Godoy M. Sánchez-Serrano J. J. Schmelz E. A. (2007). ABA is an essential signal for plant resistance to pathogens affecting JA biosynthesis and the activation of defenses in *Arabidopsis*. *Plant Cell* 19 1665–1681. 10.1105/tpc.106.048041 17513501 PMC1913739

[B2] Akita M. Valkonen J. P. (2002). A novel gene family in moss (*Physcomitrella patens*) shows sequence homology and a phylogenetic relationship with the TIR-NBS class of plant disease resistance genes. *J. Mol. Evol.* 55 595–605. 10.1007/s00239-002-2355-8 12399933

[B3] Alvarez A. Montesano M. Schmelz E. Ponce de León I. (2016). Activation of shikimate, phenylpropanoid, oxylipins, and auxin pathways in *Pectobacterium carotovorum* elicitors-treated moss. *Front. Plant Sci.* 7:328 10.3389/fpls.2016.00328 PMC4801897 27047509

[B4] Andersson R. A. Akita M. Pirhonen M. Gammelgård E. Valkonen J. P. T. (2005). Moss-Erwinia pathosystem reveals possible similarities in pathogenesis and pathogen defense in vascular and nonvascular plants. *J. Gen. Plant Pathol.* 71 23–28. 10.1007/s10327-004-0154-3

[B5] Anterola A. Göbel C. Hornung E. Sellhorn G. Feussner I. Grimes H. (2009). *Physcomitrella patens* has lipoxygenases for both eicosanoid and octadecanoid pathways. *Phytochemistry* 70 40–52. 10.1016/j.phytochem.2008.11.012 19131081

[B6] Bay G. Nahar N. Oubre M. Whitehouse M. J. Wardle D. A. Zackrisson O. (2013). Boreal feather mosses secrete chemical signals to gain nitrogen. *New Phytol.* 200 54–60. 10.1111/nph.12403 23795916

[B7] Bethke G. Pecher P. Eschen-Lippold L. Tsuda K. Katagiri F. Glazebrook J. (2012). Activation of the *Arabidopsis thaliana* mitogen-activated protein kinase MPK11 by the flagellin-derived elicitor peptide, flg22. *Mol. Plant Microbe Interact.* 25 471–480. 10.1094/MPMI-11-11-0281 22204645

[B8] Bindschedler L. Dewdney J. Blee K. Stone J. Asai T. Plotnikov J. (2006). Peroxidase-dependent apoplastic oxidative burst in Arabidopsis required for pathogen resistance. *Plant J.* 47 851–863. 10.1111/j.1365-313X.2006.02837.x 16889645 PMC3233234

[B9] Boller T. Felix G. (2009). A renaissance of elicitors: perception of microbe-associated molecular patterns and danger signals by pattern-recognition receptors. *Annu. Rev. Plant Biol.* 60 379–406. 10.1146/annurev.arplant.57.032905.105346 19400727

[B10] Boller T. He S. Y. (2009). Innate immunity in plants: an arms race between pattern recognition receptors in plants and effectors in microbial pathogens. *Science* 324 742–744. 10.1126/science.1171647 19423812 PMC2729760

[B11] Bouarab K. Adas F. Gaquerel E. Kloareg B. Salaün J. P. Potin P. (2004). The innate immunity of a marine red alga involves oxylipins from both the eicosanoid and octadecanoid pathways. *Plant Physiol.* 135 1838–1848. 10.1104/pp.103.037622 15247395 PMC519094

[B12] Bressendorff S. (2012). *Immunity in the Moss Physcomitrella Patens.* Ph.D. thesis, Department of Biology, Faculty of Science, University of Copenhagen, Copenhagen.

[B13] Bressendorff S. Azevedo R. Kenchappa C. S. Ponce de León I. Olsen J. V. Rasmussen M. W. (2016). An innate immunity pathway in the moss *Physcomitrella patens*. *Plant Cell* 28 1328–1342. 10.1105/tpc.15.00774 27268428 PMC4944399

[B14] Broekaert W. F. Delauré S. L. De Bolle M. F. Cammue B. P. (2006). The role of ethylene in host-pathogen interactions. *Annu. Rev. Phytopathol.* 44 393–416. 10.1146/annurev.phyto.44.070505.143440 16602950

[B15] Cao Y. Liang Y. Tanaka K. Nguyen C. T. Jedrzejczak R. P. Joachimiak A. (2014). The kinase LYK5 is a major chitin receptor in *Arabidopsis* and forms a chitin-induced complex with related kinase CERK1. *Elife* 23 3 10.7554/eLife.03766 PMC4356144 25340959

[B16] Caplan J. L. Kumar A. S. Park E. Padmanabhan M. S. Hoban K. Modla S. (2015). Chloroplast stromules function during innate immunity. *Dev. Cell* 34 45–57. 10.1016/j.devcel.2015.05.011 26120031 PMC4596411

[B17] Castro A. Vidal S. Ponce de León I. (2016). Moss pathogenesis-related-10 protein enhances resistance to *Pythium irregulare* in *Physcomitrella patens* and *Arabidopsis thaliana*. *Front. Plant Sci.* 7:580 10.3389/fpls.2016.00580 PMC4850436 27200053

[B18] Chico J. M. Chini A. Fonseca S. Solano R. (2008). JAZ repressors set the rhythm in jasmonate signaling. *Curr. Opin. Plant Biol.* 11 486–494. 10.1016/j.pbi.2008.06.003 18653378

[B19] Chini A. Fonseca S. Fernández G. Adie B. Chico J. M. Lorenzo O. (2007). The JAZ family of repressors is the missing link in jasmonate signalling. *Nature* 448 666–671. 10.1038/nature06006 17637675

[B20] Couto D. Zipfel C. (2016). Regulation of pattern recognition receptor signalling in plants. *Nat. Rev. Immunol.* 16 537–552. 10.1038/nri.2016.77 27477127

[B21] Cuming A. C. Stevenson S. R. (2015). From pond slime to rain forest: the evolution of ABA signalling and the acquisition of dehydration tolerance. *New Phytol.* 206 5–7. 10.1111/nph.13333 25711244

[B22] Davey M. L. Tsuneda A. Currah R. S. (2009). Pathogenesis of bryophyte hosts by the ascomycete *Atradidymella muscivora*. *Am. J. Bot.* 96 1274–1280. 10.3732/ajb.0800239 21628276

[B23] Davey M. L. Tsuneda A. Currah R. S. (2010). Saprobic and parasitic interactions of *Coniochaeta velutina* with mosses. *Botany* 88 258–265. 10.1139/B10-004

[B24] Delaux P. M. Radhakrishnan G. V. Jayaraman D. Cheema J. Malbreil M. Volkening J. D. (2015). Algal ancestor of land plants was preadapted for symbiosis. *Proc. Natl. Acad. Sci. U.S.A.* 112 13390–13395. 10.1073/pnas.1515426112 26438870 PMC4629359

[B25] Delaux P. M. Séjalon-Delmas N. Bécard G. Ané J. M. (2013). Evolution of the plant-microbe symbiotic ‘toolkit’. *Trends Plant Sci.* 18 298–304. 10.1016/j.tplants.2013.01.008 23462549

[B26] DeLuca T. H. Zackrisson O. Nilsson M. C. Sellstedt A. (2002). Quantifying nitrogen-fixation in feather moss carpets of boreal forests. *Nature* 419 917–920. 10.1038/nature01051 12410308

[B27] Denancé N. Sánchez-Vallet A. Goffner D. Molina A. (2013). Disease resistance or growth: the role of plant hormones in balancing immune responses and fitness costs. *Front. Plant Sci.* 4:155 10.3389/fpls.2013.00155 PMC3662895 23745126

[B28] Dixon R. A. Paiva N. L. (1995). Stress-induced phenylpropanoid metabolism. *Plant Cell* 7 1085–1097. 10.1105/tpc.7.7.1085 12242399 PMC160915

[B29] Emiliani G. Fondi M. Fani R. Gribaldo S. (2009). A horizontal gene transfer at the origin of phenylpropanoid metabolism: a key adaptation of plants to land. *Biol. Direct* 4:7 10.1186/1745-6150-4-7 PMC2657906 19220881

[B30] Erxleben A. Gessler A. Vervliet-Scheebaum M. Reski R. (2012). Metabolite profiling of the moss *Physcomitrella patens* reveals evolutionary conservation of osmoprotective substances. *Plant Cell Rep.* 31 427–436. 10.1007/s00299-011-1177-9 22038371

[B31] Fiil B. K. Petersen K. Petersen M. Mundy J. (2009). Gene regulation by MAP kinase cascades. *Curr. Opin. Plant Biol.* 12 615–621. 10.1016/j.pbi.2009.07.017 19716758

[B32] Fu Z. Q. Yan S. Saleh A. Wang W. Ruble J. Oka N. (2012). NPR3 and NPR4 are receptors for the immune signal salicylic acid in plants. *Nature* 486 228–232. 10.1038/nature11162 22699612 PMC3376392

[B33] Galindo-Trigo S. Gray J. E. Smith L. M. (2016). Conserved roles of CrRLK1L Receptor-Like Kinases in cell Expansion and reproduction from algae to angiosperms. *Front. Plant Sci.* 7:1269 10.3389/fpls.2016.01269 PMC5002434 27621737

[B34] Gao M. Liu J. Bi D. Zhang Z. Cheng F. Chen S. (2008). MEKK1 MKK1/MKK2 and MPK4 function together in a mitogen-activated protein kinase cascade to regulate innate immunity in plants. *Cell Res.* 18 1190–1198. 10.1038/cr.2008.300 18982020

[B35] Glazebrook J. (2005). Contrasting mechanisms of defense against biotrophic and necrotrophic pathogens. *Annu. Rev. Phytopathol.* 43 205–227. 10.1146/annurev.phyto.43.040204.135923 16078883

[B36] Gómez-Gómez L. Boller T. (2000). FLS2: an LRR receptor-like kinase involved in the perception of the bacterial elicitor flagellin in Arabidopsis. *Mol. Cell* 5 1003–1011. 10.1016/S1097-2765(00)80265-8 10911994

[B37] Gonneau M. Pagant S. Brun F. Laloue M. (2001). Photoaffinity labelling with the cytokinin agonist azido-CPPU of a 34 kDa peptide of the intracellular pathogenesis-related protein family in the moss *Physcomitrella patens*. *Plant Mol. Biol.* 46 539–548. 10.1023/A:1010693213437 11516147

[B38] Govrin E. M. Levine A. (2000). The hypersensitive response facilitates plant infection by the necrotrophic pathogen *Botrytis cinerea*. *Curr. Biol.* 10 751–757. 10.1016/S0960-9822(00)00560-1 10898976

[B39] Groen S. C. Whiteman N. K. (2014). The evolution of ethylene signaling in plant chemical ecology. *J. Chem. Ecol.* 40 700–716. 10.1007/s10886-014-0474-5 24997626

[B40] Gustavsson S. Lebrun A. S. Nordén K. Chaumont F. Johanson U. (2005). A novel plant major intrinsic protein in *Physcomitrella patens* most similar to bacterial glycerol channels. *Plant Physiol.* 139 287–295. 10.1104/pp.105.063198 16113222 PMC1203378

[B41] Hayashi K. Kawaide H. Notomi M. Sakigi Y. Matsuo A. Nozaki H. (2006). Identification and functional analysis of bifunctional ent-kaurene synthase from the moss *Physcomitrella patens*. *FEBS Lett.* 580 6175–6181. 10.1016/j.febslet.2006.10.018 17064690

[B42] Humphreys C. P. Franks P. J. Rees M. Bidartondo M. I. Leake J. R. Beerling D. J. (2010). Mutualistic mycorrhiza-like symbiosis in the most ancient group of land plants. *Nat. Commun.* 1 103 10.1038/ncomms1105 21045821

[B43] Hwang I. S. Hwang B. K. (2011). The pepper mannose-binding lectin gene CaMBL1 is required to regulate cell death and defense responses to microbial pathogens. *Plant Physiol.* 155 447–463. 10.1104/pp.110.164848 21205632 PMC3075774

[B44] Jones J. D. G. Dangl J. L. (2006). The plant immune system. *Nature* 444 323–329. 10.1038/nature05286 17108957

[B45] Keinath N. F. Kierszniowska S. Lorek J. Bourdais G. Kessler S. A. Shimosato Asano H. (2010). PAMP (pathogen-associated molecular pattern)-induced changes in plasma membrane compartmentalization reveal novel components of plant immunity. *J. Biol. Chem.* 285 39140–39149. 10.1074/M110.160531 20843791 PMC2998143

[B46] Khraiwesh B. Qudeimat E. Thimma M. Chaiboonchoe A. Jijakli K. Alzahmi A. (2015). Genome-wide expression analysis offers new insights into the origin and evolution of *Physcomitrella patens* stress response. *Sci. Rep.* 5:17434 10.1038/srep17434 PMC4663497 26615914

[B47] Kobaru S. Tanaka R. Taira T. Uchiumi T. (2016). Functional analyses of chitinases in the moss *Physcomitrella patens*: chitin oligosaccharide-induced gene expression and enzymatic characterization. *Biosci. Biotechnol. Biochem.* 80 2347–2356. 10.1080/09168451.2016.1224640 27562231

[B48] Koduri P. K. Gordon G. S. Barker E. I. Colpitts C. C. Ashton N. W. Suh D. Y. (2010). Genome-wide analysis of the chalcone synthase superfamily genes of *Physcomitrella patens*. *Plant Mol. Biol.* 72 247–263. 10.1007/s11103-009-9565-z 19876746

[B49] Lang D. Eisinger J. Reski R. Rensing S. A. (2005). Representation and high-quality annotation of the *Physcomitrella patens* transcriptome demonstrates a high proportion of proteins involved in metabolism in mosses. *Plant Biol. (Stuttg).* 7 238–250. 10.1055/s-2005-837578 15912443

[B50] Lawton M. Saidasan H. (2009). Pathogenesis in mosses. *Annu. Plant Rev.* 36 298–339.

[B51] Lehti-Shiu M. D. Zou C. Hanada K. Shiu S. H. (2009). Evolutionary history and stress regulation of plant receptor-like kinase/pelle genes. *Plant Physiol.* 150 12–26. 10.1104/pp.108.134353 19321712 PMC2675737

[B52] Lehtonen M. T. Akita M. Frank W. Reski R. Valkonen J. P. (2012a). Involvement of a class III peroxidase and the mitochondrial protein TSPO in oxidative burst upon treatment of moss plants with a fungal elicitor. *Mol. Plant Microbe Interact.* 25 363–371. 10.1094/MPMI-10-11-0265 22112216

[B53] Lehtonen M. T. Akita M. Kalkkinen N. Ahola-Iivarinen E. Rönnholm G. Somervuo P. (2009). Quickly-released peroxidase of moss in defense against fungal invaders. *New Phytol.* 183 432–443. 10.1111/j.1469-8137.2009.02864.x 19453432

[B54] Lehtonen M. T. Marttinen E. M. Akita M. Valkonen J. P. T. (2012b). Fungi infecting cultivated moss can also cause diseases in crop plants. *Ann. Appl. Biol.* 160 298–307. 10.1111/j.1744-7348.2012.00543.x

[B55] Lehtonen M. T. Takikawa Y. Rönnholm G. Akita M. Kalkkinen N. Ahola-Iivarinen E. (2014). Protein secretome of moss plants (*Physcomitrella patens*) with emphasis on changes induced by a fungal elicitor. *J. Proteome Res.* 13 447–459. 10.1021/pr400827a 24295333

[B56] Lenz H. D. Haller E. Melzer E. Kober K. Wurster K. Stahl M. (2011). Autophagy differentially controls plant basal immunity to biotrophic and necrotrophic pathogens. *Plant J.* 66 818–830. 10.1111/j.1365-313X.2011.04546.x 21332848

[B57] Li J. L. Sulaiman M. Beckett R. P. Minibayeva F. V. (2010). Cell wall peroxidases in the liverwort Dumortiera hirsute are responsible for extracellular superoxide production and can display tyrosinase activity. *Physiol. Plant.* 138 474–484. 10.1111/j.1399-3054.2009.01318.x 19947974

[B58] Ligrone R. Carafa A. Duckett J. G. RenzagliaK K. S. Ruel K. (2008). Immunocytochemical detection of lignin-related epitopes in cell walls in bryophytes and the charalean alga Nitella. *Plant Syst. Evol.* 270 257 10.1007/s00606-007-0617-z

[B59] Lindner H. Müller L. M. Boisson-Dernier A. Grossniklaus U. (2012). CrRLK1L receptor-like kinases: not just another brick in the wall. *Curr. Opin. Plant Biol.* 6 659–669. 10.1016/j.pbi.2012.07.003 22884521

[B60] Lopez-Obando M. Hoffmann B. Géry C. Guyon-Debast A. Téoulé E. Rameau C. (2016). Simple and efficient targeting of multiple genes through CRISPR-Cas9 in *Physcomitrella patens*. *G*3 (Bethesda) 10.1534/g3.116.033266 [Epub ahead of print]. PMC5100863 27613750

[B61] Luna E. Pastor V. Robert J. Flors V. Mauch-Mani B. Ton J. (2011). Callose deposition: a multifaceted plant defense response. *Mol. Plant Microbe Interact.* 24 183–193. 10.1094/MPMI-07-10-0149 20955078

[B62] Machado L. Castro A. Hamberg M. Bannenberg G. Gaggero C. Castresana C. (2015). The *Physcomitrella patens* unique alpha-dioxygenase participates in both developmental processes and defense responses. *BMC Plant Biol.* 15:439 10.1186/s12870-015-0439-z PMC4334559 25848849

[B63] Meeks J. C. Elhai J. (2002). Regulation of cellular differentiation in filamentous cyanobacteria in free-living and plant-associated symbiotic growth states. *Microbiol. Mol. Biol. Rev.* 66 94–121. 10.1128/MMBR.66.1.94-121.2002 11875129 PMC120779

[B64] Meng X. Zhang S. (2013). MAPK cascades in plant disease resistance signaling. *Annu. Rev. Phytopathol.* 51 245–266. 10.1146/annurev-phyto-082712-102314 23663002

[B65] Mikami K. Hartmann E. (2004). “Lipid metabolism in mosses,” in *New Frontiers in Bryology: Physiology, Molecular Biology and Functional Genomics*, eds Wood A. J. Oliver M. J. Cove D. J. (Dordrecht: Kluwer Academic Publishers), 133–155.

[B66] Mittag J. Šola I. Rusak G. Ludwig-Müller J. (2015). *Physcomitrella patens* auxin conjugate synthetase (GH3) double knockout mutants are more resistant to Pythium infection than wild type. *J. Plant Physiol.* 183 75–83. 10.1016/j.jplph.2015.05.015 26102574

[B67] Miya A. Albert P. Shinya T. Desaki Y. Ichimura K. Shirasu K. (2007). CERK1 a LysM receptor kinase, is essential for chitin elicitor signaling in *Arabidopsis*. *Proc. Natl. Acad. Sci. U.S.A.* 104 19613–19618. 10.1073/pnas.0705147104 18042724 PMC2148337

[B68] Miyazaki S. Nakajima M. Kawaide H. (2015). Hormonal diterpenoids derived from ent-kaurenoic acid are involved in the blue-light avoidance response of *Physcomitrella patens*. *Plant Signal. Behav.* 10:e989046 10.4161/15592324.2014.989046 PMC4622475 25751581

[B69] Mukae K. Inoue Y. Moriyasu Y. (2015). ATG5-knockout mutants of *Physcomitrella* provide a platform for analyzing the involvement of autophagy in senescence processes in plant cells. *Plant Signal. Behav.* 10:e1086859 10.1080/15592324.2015.1086859 PMC4883962 26368055

[B70] Mukherjee A. (2015). Computational study of a bifunctional ent-kaurene synthase from *Physcomitrella patens* (Hedw.) Bruch & Schimp.: an insight into the origin of terpenoid biosynthesis in plants. *Acta Bot. Gall.* 162 139–152. 10.1080/12538078.2015.1017774

[B71] Ngaki M. N. Louie G. V. Philippe R. N. Manning G. Pojer F. Bowman M. E. (2012). Evolution of the chalcone-isomerase fold from fatty-acid binding to stereospecific catalysis. *Nature* 485 530–533. 10.1038/nature11009 22622584 PMC3880581

[B72] Nissen K. S. Willats W. G. Malinovsky F. G. (2016). Understanding CrRLK1L function: cell walls and growth control. *Trends Plant Sci.* 21 516–527. 10.1016/j.tplants.2015.12.004 26778775

[B73] Okada K. Kawaide H. Miyamoto K. Miyazaki S. Kainuma R. Kimura H. (2016). HpDTC1 a stress-inducible bifunctional diterpene cyclase involved in momilactone biosynthesis, functions in chemical defence in the moss *Hypnum plumaeforme*. *Sci. Rep.* 6:25316 10.1038/srep25316 PMC4853780 27137939

[B74] Oliver J. P. Castro A. Gaggero C. Cascón T. Schmelz E. A. Castresana C. (2009). Pythium infection activates conserved plant defense responses in mosses. *Planta* 230 569–579. 10.1007/s00425-009-0969-4 19551405

[B75] Opelt K. Berg C. Berg G. (2007a). The bryophyte genus Sphagnum is a reservoir for powerful and extraordinary antagonists and potentially facultative human pathogens. *FEMS Microbiol. Ecol.* 61 38–53. 10.1111/j.1574-6941.2007.00323.x 17484734

[B76] Opelt K. Berg C. Schönmann S. Eberl L. Berg G. (2007b). High specificity but contrasting biodiversity of Sphagnum-associated bacterial and plant communities in bog ecosystems independent of the geographical region. *ISME J.* 1 502–516. 10.1038/ismej.2007.58 18043652

[B77] Overdijk E. J. De Keijzer J. De Groot D. Schoina C. Bouwmeester K. Ketelaar T. (2016). Interaction between the moss *Physcomitrella patens* and *Phytophthora*: a novel pathosystem for live-cell imaging of subcellular defence. *J. Microsc.* 263 171–180. 10.1111/jmi.12395 27027911

[B78] Pérez-Pérez M. E. Lemaire S. D. Crespo J. L. (2012). Reactive oxygen species and autophagy in plants and algae. *Plant Physiol.* 160 156–164. 10.1104/pp.112.199992 22744983 PMC3440194

[B79] Ponce de León I. (2011). The moss *Physcomitrella patens* as a model system to study interactions between plants and phytopathogenic fungi and oomycetes. *J. Pathog.* 2011:719873 10.4061/2011/719873 PMC3335576 22567339

[B80] Ponce de León I. Hamberg M. Castresana C. (2015). Oxylipins in moss development and defense. *Front. Plant Sci.* 6:483 10.3389/fpls.2015.00483 PMC4490225 26191067

[B81] Ponce de León I. Montesano M. (2013). Activation of defense mechanisms against pathogens in mosses and flowering plants. *Int. J. Mol. Sci.* 14 3178–3200. 10.3390/ijms14023178 23380962 PMC3588038

[B82] Ponce de León I. Oliver J. P. Castro A. Gaggero C. Bentancor M. Vidal S. (2007). *Erwinia carotovora* elicitores and *Botrytis cinerea* activate defense responses in *Physcomitrella patens*. *BMC Plant Biol.* 7:52 10.1186/1471-2229-7-52 PMC2174466 17922917

[B83] Ponce de León I. Schmelz E. Gaggero C. Castro A. Alvarez A. Montesano M. (2012). *Physcomitrella patens* activates reinforcement of the cell wall, programmed cell death and accumulation of evolutionary conserved defense signals like SA and OPDA but not JA upon *Botrytis cinerea* infection. *Mol. Plant Pathol.* 13 960–974. 10.1111/j.1364-703.2012.00806.x 22551417 PMC6638766

[B84] Raven J. A. (1984). Physiological correlates of the morphology of early vascular plants. *Bot. J. Linn. Soc.* 88 105–126. 10.1111/j.1095-8339.1984.tb01566.x

[B85] Read D. J. Duckett J. G. Francis R. Ligrone R. Russell A. (2000). Symbiotic fungal associations in ‘lower’ land plants. *Philos. Trans. R. Soc. Lond. B. Biol. Sci.* 355 815–830. 10.1098/rstb.2000.0617 10905611 PMC1692782

[B86] Reboledo G. Del Campo R. Alvarez A. Montesano M. Mara H. Ponce de León I. (2015). *Physcomitrella patens* activates defense responses against the pathogen *Colletotrichum gloeosporioides*. *Int. J. Mol. Sci.* 16 22280–22298. 10.3390/ijms160922280 26389888 PMC4613308

[B87] Redecker D. Kodner R. Graham L. E. (2000). Glomalean fungi from the Ordovician. *Science* 289 1920–1921. 10.1126/science.289.5486.1920 10988069

[B88] Rensing S. A. Ick J. Fawcett J. A. Lang D. Zimmer A. Van de Peer Y. (2007). An ancient genome duplication contributed to the abundance of metabolic genes in the moss *Physcomitrella patens*. *BMC Evol. Biol.* 7:130 10.1186/1471-2148-7-130 PMC1952061 17683536

[B89] Rensing S. A. Lang D. Zimmer A. D. Terry A. Salamov A. Shapiro H. (2008). The *Physcomitrella* genome reveals evolutionary insights into the conquest of land by plants. *Science* 319 64–69. 10.1126/science.1150646 18079367

[B90] Rodriguez M. C. Petersen M. Mundy J. (2010). Mitogen activated protein kinase signaling in plants. *Annu. Rev. Plant Biol.* 61 621–649. 10.1146/annurev-arplant-042809-112252 20441529

[B91] Sarris P. F. Cevik V. Dagdas G. Jones J. D. Krasileva K. V. (2016). Comparative analysis of plant immune receptor architectures uncovers host proteins likely targeted by pathogens. *BMC Biol.* 14:8 10.1186/s12915-016-0228-7 PMC4759884 26891798

[B92] Schaefer D. G. (2002). A new moss genetics: targeted mutagenesis in *Physcomitrella patens*. *Annu. Rev. Plant Biol.* 53 477–501. 10.1146/annurev.arplant.53.100301.135202 12221986

[B93] Senger T. Wichard T. Kunze S. Göbel C. Lerchl J. Pohnert G. (2005). A multifunctional lipoxygenase with fatty acid hydroperoxide cleaving activity from the moss *Physcomitrella patens*. *J. Biol. Chem.* 280 7588–7596. 10.1074/jbc.M411738200 15611050

[B94] Seybold H. Trempel F. Ranf S. Scheel D. Romeis T. Lee J. (2014). Ca2+ signalling in plant immune response: from pattern recognition receptors to Ca*2*+ decoding mechanisms. *New Phytol.* 204 782–790. 10.1111/nph.13031 25539002

[B95] Strullu-Derrien C. Kenrick P. Pressel S. Duckett J. G. Rioult J. P. Strullu D. G. (2014). Fungal associations in *Horneophyton ligneri* from the Rhynie Chert (c. 407 million year old) closely resemble those in extant lower land plants: novel insights into ancestral plant-fungus symbioses. *New Phytol.* 203 964–979. 10.1111/nph.12805 24750009

[B96] Takemoto D. Jones D. A. Hardham A. R. (2006). Re-organization of the cytoskeleton and endoplasmic reticulum in the Arabidopsis pen1-1 mutant inoculated with the non-adapted powdery mildew pathogen, *Blumeria graminis* f. sp. hordei. *Mol. Plant Pathol.* 7 553–563. 10.1111/j.1364-3703.2006.00360.x 20507469

[B97] Taki N. Sasaki-Sekimoto Y. Obayashi T. Kikuta A. Kobayashi K. Ainai T. (2005). 12-oxo-phytodienoic acid triggers expression of a distinct set of genes and plays a role in wound-induced gene expression in Arabidopsis. *Plant Physiol.* 139 1268–1283. 10.1104/pp.105.067058 16258017 PMC1283764

[B98] Tambalo D. D. Vanderlinde E. M. Robinson S. Halmillawewa A. Hynes M. F. Yost C. K. (2014). Legume seed exudates and *Physcomitrella patens* extracts influence swarming behavior in *Rhizobium leguminosarum*. *Can. J. Microbiol.* 60 15–24. 10.1139/cjm-2013-0723 24392922

[B99] Tang J. Y. Ma J. Li X. D. Li Y. H. (2016). Illumina sequencing-based community analysis of bacteria associated with different bryophytes collected from Tibet, China. *BMC Microbiol.* 16:276 10.1186/s12866-016-0892-3 PMC5112639 27852238

[B100] Tanigaki Y. Ito K. Obuchi Y. Kosaka A. Yamato K. T. Okanami M. (2014). *Physcomitrella patens* has kinase-LRR R gene homologs and interacting proteins. *PLoS ONE* 9:e95118 10.1371/journal.pone.0095118 PMC3991678 24748046

[B101] Torres M. A. Dangl J. L. Jones J. D. G. (2002). Arabidopsisg p91phox homologues AtrbohD and AtrbohF are required for accumulation of reactive oxygen intermediates in the plant defense response. *Proc. Natl. Acad. Sci. U.S.A.* 99 517–522. 10.1073/pnas.012452499 11756663 PMC117592

[B102] Torres M. A. Jones J. D. Dangl J. L. (2006). Reactive oxygen species signaling in response to pathogens. *Plant Physiol.* 141 373–378. 10.1104/pp.106.079467 16760490 PMC1475467

[B103] van Loon L. C. Rep M. Pietersen C. M. J. (2006). Significance of inducible defense-related proteins in infected plants. *Annu. Rev. Phytopathol.* 44 135–162. 10.1146/annurev.phyto.44.070505.143425 16602946

[B104] Von Schwartzenberg K. Schultze W. Kassner H. (2004). The moss *Physcomitrella patens* releases a tetracyclic diterpene. *Plant Cell Rep.* 22 780–786. 10.1007/s00299-004-0754-6 14963693

[B105] Wan J. Tanaka K. Zhang X. C. Son G. H. Brechenmacher L. Nguyen T. H. (2012). LYK4 a lysin motif receptor-like kinase, is important for chitin signaling and plant innate immunity in *Arabidopsis*. *Plant Physiol.* 160 396–406. 10.1104/pp.112.201699 22744984 PMC3440214

[B106] Wang C. Liu Y. Li S.-S. Han G.-Z. (2015). Insights into the origin and evolution of the plant hormone signaling machinery. *Plant Physiol.* 167 872–886. 10.1104/pp.114.247403 25560880 PMC4348752

[B107] Wang X. Kuang T. He Y. (2010). Conservation between higher plants and the moss *Physcomitrella patens* in response to the phytohormone abscisic acid: a proteomics analysis. *BMC Plant Biol.* 10:192 10.1186/1471-2229-10-192 PMC2956542 20799958

[B108] Wei Y. Shen W. Dauk M. Wang F. Selvaraj G. Zou J. (2004). Targeted gene disruption of glycerol-3-phosphate dehydrogenase in *Colletotrichum gloeosporioides* reveals evidence that glycerol is a significant transferred nutrient from host plant to fungal pathogen. *J. Biol. Chem.* 279 429–435. 10.1074/jbc.M308363200 14563847

[B109] Wichard T. Göbel C. Feussner I. Pohnert G. (2005). Unprecedented lipoxygenase/hydroperoxide lyase pathways in the moss *Physcomitrella patens*. *Angew. Chem. Int. Ed.* 44 158–161. 10.1002/anie.200460686 15599905

[B110] Winter P. S. Bowman C. E. Villani P. J. Dolan T. E. Hauck N. R. (2014). Systemic acquired resistance in moss: further evidence for conserved defense mechanisms in plants. *PLoS ONE* 9:e101880 10.1371/journal.pone.0101880 PMC4085009 25000589

[B111] Wolf L. Rizzini L. Stracke R. Ulm R. Rensing S. A. (2010). The molecular and physiological responses of *Physcomitrella patens* to ultraviolet-B radiation. *Plant Physiol.* 153 1123–1134. 10.1104/pp.110.154658 20427465 PMC2899899

[B112] Xue J.-Y. Wang Y. Wu P. Wang Q. Yang L.-T. Pan X.-H. (2012). A primary survey on bryophyte species reveals two novel classes of nucleotide-binding site (NBS) genes. *PLoS ONE* 7:e36700 10.1371/journal.pone.0036700 PMC3352924 22615795

[B113] Yamamoto Y. Ohshika J. Takahashi T. Ishizaki K. Kohchi T. Matusuura H. (2015). Functional analysis of allene oxide cyclase, MpAOC, in the liverwort *Marchantia polymorpha*. *Phytochemistry* 116 48–56. 10.1016/j.phytochem.2015.03.008 25892411

[B114] Yasumura Y. Crumpton-Taylor M. Fuentes S. Harberd N. P. (2007). Step-by-step acquisition of the gibberellin-DELLA growth-regulatory mechanism during land-plant evolution. *Curr. Biol.* 17 1225–1230. 10.1016/j.cub.2007.06.037 17627823

[B115] Yasumura Y. Pierik R. Fricker M. D. Voesenek L. A. Harberd N. P. (2012). Studies of *Physcomitrella patens* reveal that ethylene-mediated submergence responses arose relatively early in land-plant evolution. *Plant J.* 72 947–959. 10.1111/tpj.12005 23046428

[B116] Yue J. Hu X. Sun H. Yang Y. Huang J. (2012). Widespread impact of horizontal gene transfer on plant colonization of land. *Nat. Commun.* 3:1152 10.1038/ncomms2148 PMC3493653 23093189

[B117] Zhang L. Du L. Poovaiah B. W. (2014). Calcium signaling and biotic defense responses in plants. *Plant Signal. Behav.* 9:e973818 10.4161/15592324.2014.973818 PMC4623097 25482778

[B118] Zimmer A. D. Lang D. Buchta K. Rombauts S. Nishiyama T. Hasebe M. (2013). Reannotation and extended community resources for the genome of the non-seed plant *Physcomitrella patens* provide insights into the evolution of plant gene structures and functions. *BMC Genomics* 14:498 10.1186/1471-2164-14-498 PMC3729371 23879659

[B119] Zipfel C. (2009). Early molecular events in PAMP-triggered immunity. *Curr. Opin. Plant Biol.* 12 414–420. 10.1016/j.pbi.2009.06.003 19608450

[B120] Zipfel C. Kunze G. Chinchilla D. Caniard A. Jones J. D. G. Boller T. (2006). Perception of the bacterial PAMP EF-Tu by the receptor EFR restricts Agrobacterium-mediated transformation. *Cell* 125 749–760. 10.1016/j.cell.2006.03.037 16713565

